# Urinary metabolomics and proteomics for early detection of gastric cancer: insights from a two-center multicenter study

**DOI:** 10.3389/fonc.2026.1733804

**Published:** 2026-04-01

**Authors:** Yadan Wang, Jing Wu, Jiayi Su, Wenkun Li, Pengpeng Ding, Miaomiao Wang

**Affiliations:** 1 Department of Gastroenterology, Beijing Friendship Hospital, Capital Medical University, National Clinical Research Center for Digestive Diseases, Beijing Digestive Disease Center, Beijing Key Laboratory for Precancerous Lesion of Digestive Diseases, Beijing, China; 2 Department of Gastroenterology, Beijing Shijitan Hospital, Capital Medical University, Beijing, China

**Keywords:** biomarkers, diagnostic model, gastric cancer, metabolomics, proteomics

## Abstract

**Aims:**

This study aimed to develop a non-invasive method combining urinary metabolomics and proteomics to identify biomarkers for gastric cancer and to elucidate the molecular mechanisms associated with its progression.

**Methods:**

Urine samples from 30 advanced gastric cancer (AGC), 30 early gastric cancer (EGC), and 30 healthy controls (CG) were collected at two centers. Differential metabolites were identified using the UHPLC-MS instrument (VIP>1, FDR<0.05, |log2FC|≥1), and proteins were quantified using the TMT-based proteomics approach (VIP>1, FDR<0.05, |log2FC| ≥ 1.2). Key metabolites were selected via Random Forest and Boruta algorithms, and proteomic findings were validated using TCGA data. Gene Ontology (GO) and Kyoto Encyclopedia of Genes and Genomes (KEGG) analyses were performed to explore enriched pathways.

**Results:**

A total of 350 differential metabolites were identified in AGC vs. CG, and 285 in EGC vs. CG, primarily involving amino acid, bile acid, and energy metabolism. Core metabolites such as butyrate, indolelactic acid, D-ribose-5-phosphate, and serine were included in diagnostic models. Proteomic analysis identified 376 differentially abundant proteins in AGC and 191 in EGC, enriched in immune response, cell adhesion, and protein hydrolysis. Key proteins in AGC included TNFRSF12A, ITGB3, HSPA8, and FTL, with significant upregulation or downregulation observed for each. For example, TNFRSF12A was upregulated (p < 0.05), while HSPA8 was downregulated (p < 0.05), and these proteins were linked to pathways such as cell adhesion molecules (CAMs), ECM–receptor interaction, and platelet activation. In EGC, proteins like ITGB3 and FTL were significantly upregulated (p < 0.05), with involvement in pathways such as HIF-1 signaling, glycolysis/gluconeogenesis, and antigen processing/presentation. Integrated analysis revealed 43 significantly enriched KEGG pathways in AGC and 30 in EGC, with notable pathways in amino acid metabolism, the TCA cycle, PI3K-Akt signaling, and immune response pathways. These findings highlight the involvement of cell adhesion, immune response, and metabolic signaling in the pathobiology of gastric cancer.

**Conclusion:**

The combination of urinary metabolomics and proteomics enables non-invasive detection of gastric cancer, revealing key biomarkers and pathways with potential clinical diagnostic significance. Further investigation is needed to confirm the diagnostic value of these findings in clinical practice.

## 1 Introduction

Gastric cancer (GC) remains a major malignancy of the digestive tract worldwide. According to the World Health Organization, over 1.0 million new cases and approximately 769,000 deaths occurred in 2020, with China contributing 479,000 cases and 374,000 deaths-accounting for 44.0% and 48.6% of global totals, respectively. This positions GC as the fifth most common cancer and the fourth leading cause of cancer mortality globally (1). Survival rates are stage-dependent, with over 90% survival at stage I but only around 15% at stage IV with distant metastasis (2). Thus, early detection and accurate diagnosis are critical. While endoscopy with histopathology is the diagnostic gold standard, its invasiveness, cost, and procedural complexity limit widespread screening. Several biofluids, including serum, plasma, and gastric juice, have been investigated for potential biomarkers (3–5), but commonly used serum markers such as CEA, CA19-9, and CA72–4 demonstrate suboptimal sensitivity and positive predictive value (6–8), highlighting the need for more reliable biomarkers to reduce GC incidence and mortality.

Urine has gained attention as a promising alternative biospecimen for cancer biomarker discovery (9). Compared to blood, urine can be collected noninvasively, allows for repeated sampling, and has a simpler proteome, making it ideal for detecting subtle molecular changes (10). Urinary proteins reflect systemic pathophysiology and tumor-related signals filtered by the kidneys, making urine-based proteomics an attractive approach for cancer detection (11). In addition to proteomics, urinary metabolomics is also increasingly recognized for its potential in identifying biomarkers for cancer. Urinary metabolites provide a comprehensive profile of systemic metabolic changes and can reflect biochemical shifts in response to tumor progression or treatment.

Urinary metabolomics and proteomics have shown growing promise in GC. Shimura et al. identified TFF1 and ADAM12 in urine, which, combined with H. pylori status, differentiated GC from controls (12). Using tandem mass tag (TMT)–based quantitative proteomics, Joshi et al. identified differentially expressed urinary proteins, including vitronectin and sortilin, and demonstrated their potential diagnostic relevance for gastric cancer using mass spectrometry-based biomarker discovery approaches (13). Fan et al. mapped urinary proteomic changes across gastric lesion progression and identified signatures predictive of GC risk (14). Likewise, urinary metabolomic profiling has identified altered metabolites associated with GC progression, offering a complementary approach for early diagnosis and monitoring. However, broad, multicenter validation remains limited.

In this study, we applied both urinary metabolomics and TMT-based quantitative proteomics to urine samples from patients with advanced GC (AGC), early-stage GC (EGC), and non-cancer controls (CG), recruited from two medical centers. We conducted comparative profiling of both metabolic and proteomic alterations to identify differentially expressed urinary metabolites and proteins, assessing their potential as noninvasive biomarkers for GC detection.

## 2 Materials and method

### 2.1 Clinical samples and sample preparation

We analyzed 90 urine specimens: 30 from patients with advanced gastric cancer (AGC), 30 from early gastric cancer (EGC), and 30 from healthy controls (CG). Clinical categorization followed the Japanese Gastric Cancer Association (2018) and the AJCC 8th TNM system (14, 15): EGC was defined as tumor limited to mucosa/submucosa (<=T1), regardless of nodal status, and AGC as invasion into the muscularis propria or deeper (>=T2). This study was approved by the ethics committees of Beijing Shijitan Hospital (approval number: SJTkyll-LX-2020(19)) and Beijing Friendship Hospital (approval number: 2022-P2-048-01).

Exclusion criteria included a history of hypertension, diabetes, coronary heart disease, systemic infection, estimated glomerular filtration rate <30 mL/min, other malignancies, and ongoing radiotherapy, chemotherapy, or other pharmacologic treatments. All participants provided written informed consent. The protocol was approved by the ethics committees of Beijing Shijitan Hospital and Beijing Friendship Hospital. Enrollment and sampling took place from September 2021 to May 2023 in China. Eligible cases were untreated men or women aged 34–80 years with AGC or EGC at diagnosis. CG participants were verified to be free of neoplasms through comprehensive assessment, including gastrointestinal endoscopy, blood testing, fecal occult blood testing, chest radiography, abdominal ultrasonography, and physical examination.

Each subject provided urine samples (30–50 mL), which were then processed for metabolomics and proteomics analysis as described below. For proteomics, a portion of urine was mixed with cold acetone to precipitate proteins (1:3). Then,

300 µL of SDT lysis buffer was added to each sample, followed by heating at 95°C for 3 minutes and sonication for 2 minutes. SDT lysis buffer was composed of 4% SDS, 100 mM Tris-HCl (pH 7.6), and 1 mM DTT. Samples were centrifuged at 16,000 g for 20 minutes at 4°C, and the supernatant was collected. Protein concentration was determined using the BCA assay following acetone precipitation and removal of insoluble components. To reduce inter-sample variability, all proteomic samples were normalized based on total protein input prior to TMT labeling. Creatinine-based normalization was not applied in this study and is acknowledged as a limitation.

For metabolomics, a separate aliquot of urine was processed using a similar procedure, but without protein precipitation. The sample was mixed with cold methanol-acetonitrile extraction solvent, sonicated for 10 minutes at 4°C, incubated for 30 minutes at -20 °C, and then centrifuged at 14,000 rpm for 10 minutes at 4°C. The supernatant was collected and dried under vacuum for LC-MS analysis. Additionally, the quality control (QC) samples were prepared separately for AGC, EGC, and CG groups to ensure consistency and reliability in the analysis.

### 2.2 Metabolomics analysis

#### 2.2.1 Sample preparation

100 µL of urine samples were thoroughly mixed with 400 µL of cold methanol-acetonitrile (1:1, v/v) using vortexing. The mixture was then subjected to sonication in an ice bath for 1 hour, followed by incubation at -20°C for 1 hour.

Samples were centrifuged at 16,000 g for 20 minutes at 4°C, and the supernatant was collected. The supernatant was then dried under vacuum for LC-MS analysis.

To ensure data quality for metabolic profiling, quality control (QC) samples were prepared by pooling aliquots of all samples, which were representative of the entire sample set. These QC samples were used for data normalization. QC samples were prepared and analyzed using the same procedure as the experimental samples in each batch. Dried extracts were dissolved in 50% acetonitrile, filtered using a disposable 0.22 µm cellulose acetate filter, transferred to 2 mL HPLC vials, and stored at -80°C until analysis.

#### 2.2.2 UHPLC-MS/MS analysis

Metabolomics profiling was conducted using a UPLC-ESI-Q-Orbitrap-MS system (Shimadzu Nexera X2 LC-30AD, Shimadzu, Japan) coupled with a Q-Exactive Plus mass spectrometer (Thermo Fisher Scientific, San Jose, USA).

For hydrophilic interaction liquid chromatography (HILIC) separation, samples were analyzed using a 2.1 mm×100 mm ACQUIY UPLC BEH Amide 1.7μm column (Waters, Ireland). The flow rate was set at 0.5 mL/min, and the mobile phase consisted of: A: 25 mM ammonium acetate and 25 mM ammonium hydroxide in water, and B: 100% acetonitrile (ACN). The gradient was as follows: 0–1 min, 95% B; 1–7 min, linearly decreased to 65% B; 7–9 min, decreased to 35% B; 9-9.5 min, increased to 90% B; followed by 2 minutes of re-equilibration. Both positive and negative electrospray ionization (ESI) modes were employed for MS data acquisition. The HESI source settings were as follows: Spray Voltage: 3.8 kV (+) and 3.2 kV (-); Capillary.

Temperature: 320°C; Sheath Gas: 30; Aux Gas: 5; Probe Heater Temperature: 350°C; S-Lens RF Level: 50. The MS data were acquired in a m/z range of 80–1200 Da, with full MS scans performed at a resolution of 70,000 at m/z 200, and MS/MS scans at a resolution of 17,500 at m/z 200. The maximum injection time was set to 100 ms for MS and 50 ms for MS/MS. The isolation window for MS2 was set to 2 m/z, with normalized collision energies of 27, 29, and 32 for fragmentation.

QC samples were injected every six samples during acquisition to monitor data quality and were used for data normalization. Blank samples (75% ACN in water) and QC samples were analyzed in each batch.

#### 2.2.3 Data preprocessing and filtering

Raw MS data were processed using MS-DIAL for peak alignment, retention time correction, and peak area extraction. The metabolites were identified by accurate mass (mass tolerance <0.02 Da) and MS/MS data (mass tolerance <0.02 Da), which were matched with public databases such as HMDB, MassBank, and our in-house metabolite standard library. In the extracted-ion features, only variables with more than 50% non-zero measurements in at least one group were retained.

#### 2.2.4 Multivariate statistical analysis

Multivariate data analyses and modeling were performed using R (version 4.0.3) and R packages. Data were mean-centered and Pareto scaled. Models were built using principal component analysis (PCA), orthogonal partial least squares discriminant analysis (OPLS-DA), and partial least squares discriminant analysis (PLS-DA). To prevent overfitting, permutation tests were conducted to evaluate the models. The model performance was assessed based on R2X (cumulative) and R2Y (cumulative), where perfect models correspond to R2X (cum) = 1 and R2Y (cum) = 1. The predictive ability of the models was evaluated using Q2 (cumulative), with a perfect model corresponding to Q2 (cum) = 1. A permutation test (n = 200) was performed, and the permuted model should not predict classes: R2 and Q2 values at the Y-axis intercept must be lower than those for the non-permuted model. OPLS-DA was used to identify discriminating metabolites based on the variable importance on projection (VIP) score. A VIP score >1.0 was considered statistically significant, with a higher score indicating a greater contribution to class discrimination.

Differential metabolites were selected based on VIP scores greater than 1.0 and p-values less than 0.05 obtained from a two-tailed Student ‘s t-test on normalized raw data. For multiple group comparisons, one-way analysis of variance (ANOVA) was performed. Fold changes were calculated as the logarithm of the ratio of the average mass response (area) between two groups. Cluster analysis of the identified differential metabolites was performed using R packages.

#### 2.2.5 KEGG enrichment analysis

Differential metabolites were subjected to KEGG pathway analysis using the KEGG database (http://www.kegg.jp). KEGG enrichment analysis was performed using Fisher’s exact test, and false discovery rate (FDR) correction for multiple testing was applied. Enriched KEGG pathways were considered statistically significant at the p < 0.05 level.

### 2.3 Proteomics analysis

#### 2.3.1 Protein digestion

Digestion of protein (200 μ g for each sample) was performed according to the FASP procedure described by Wisniewski, Zougman et al. (15). Briefly, the detergent, DTT and other low-molecular-weight components were removed using 200 μl UA buffer (8 M Urea, 150 mM Tris-HCl pH 8.0) by repeated ultrafiltration (Microcon units, 30 kD) facilitated by centrifugation, centrifugation was performed at 14,000 rpm for 10 minutes at 4°C. Then 100 μ L 0.05 M iodoacetamide in UA buffer was added to block reduced cysteine residues and the samples were incubated for 20 min in darkness. The filter was washed with 100 μl UA buffer three times and then 100 μl 25 mM NH4HCO3 twice. Finally, the protein suspension was digested with 4 μg trypsin (Promega) in 40 μl 25 mM NH4HCO3 overnight at 37°C, and the resulting peptides were collected as a filtrate. The peptide concentration was determined with OD280 by Nanodrop device.

#### 2.3.2 TMT labeling of peptides

Peptides were labeled with TMT reagents according to the manufacturer ‘ s instructions (Thermo Fisher Scientific). Each aliquot (100 μ g of peptide equivalent) was reacted with one tube of TMT reagent, respectively. After the protein extract was dissolved in 100 μL of 0.05M TEAB solution (pH 8.5), the TMT reagent was dissolved in 41 μL of anhydrous acetonitrile and then added to the protein extract for labeling. The mixture was incubated at room temperature for 1 h. Then 8 μL of 5% hydroxylamine to the sample and incubate for 15 minutes to quench the reaction. The Multiplex labeled samples were pooled together and lyophilized.

#### 2.3.3 High pH reverse phase fractionation

TMT-labeled peptides mixture was fractionated using a Waters XBridge BEH130 column (C18, 3.5 μ m, 2.1 × 150mm) on a Agilent 1290 HPLC operating at 0.3 mL/min. Buffer A consisted of 10mM ammonium formate and buffer B consisted of 10mM ammonium formate with 90% acetonitrile; both buffers were adjusted to pH 10 with ammonium hydroxide. A total of 30 fractions were collected for each peptides mixture, and then concatenated to 15 (pooling equal interval RPLC fractions). The fractions were dried for nano LC-MS/MS analysis.

#### 2.3.4 LC-MS analysis (TMT10plex)

LC- MS analysis were performed on a Q Exactive mass spectrometer that was coupled to Easy nLC (Thermo Fisher Scientific). Peptide from each fraction was loaded onto a the C18-reversed phase column (12 cm long, 75 μm ID, 3 μm) in buffer A (2% acetonitrile and 0. 1% Formic acid) and separated with a linear gradient of buffer B (90% acetonitrile and 0. 1% Formic acid) at a flow rate of 300 nL/min over 90 min. The linear gradient was set as follows: 0–2 min, linear gradient from 2% to 5% buffer B; 2–62 min, linear gradient from 5% to 20% buffer B; 62–80 min, linear gradient from 20% to 35% buffer B; 80–83 min, linear gradient from 35% to 90% buffer B; 83–90 min, buffer B maintained at 90%. MS data was acquired using a data-dependent top15 method dynamically choosing the most abundant precursor ions from the survey scan (300–1800 m/z) for HCD fragmentation. Determination of the target value is based on predictive Automatic Gain Control (pAGC). The AGC target values of 1e6, and maximum injection time 50 ms were for full MS, and a target AGC value of 1e5, maximum injection time 100 ms for MS2. Dynamic exclusion duration was 30s. Survey scans were acquired at a resolution of 70,000 at m/z 200 and resolution for HCD spectra was set to 35,000 at m/z 200. Normalized collision energy was 30. The instrument was run with peptide recognition mode enabled. The TMT 10-plex strategy was selected based on instrument availability and experimental consistency at the time of study initiation. Given the sample size, multiple TMT batches were required, and batch effects were minimized using pooled reference channels and post-acquisition normalization strategies.

#### 2.3.5 Identification of the differentially expressed proteins

Protein identification and quantification were carried out in Proteome Discoverer ™ 2.4 (Thermo Fisher Scientific). Searches assumed trypsin specificity with up to two missed cleavages, a precursor tolerance of ±10 ppm, and a fragment tolerance of 0.02 Da. Carbamidomethyl-Cys was set as a fixed modification and Met oxidation as variable. Spectra were queried against the UniProt human reference proteome.

Quantification used TMT 10-plex reporter ions with a global 1% FDR controlled via a target–decoy approach. Protein groups required ≥1 unique peptide and Sequest HT score >0 to be retained for quantitation. Reporter intensities were normalized within the PD workflow; inter-set variation was further reduced using a pooled reference channel and the MIX strategy.

For differential analysis, limma/MSstatsTMT with empirical-Bayes variance moderation was applied, and P values were adjusted by Benjamini–Hochberg (FDR < 0.05). To filter out negligible shifts, proteins were considered biologically meaningful only when |log2 fold-change| > 0.263 (~ 1.2-fold), a commonly used TMT threshold balancing sensitivity and interpretability. Results remained consistent under a stricter cutoff of 0.378 (~ 1.3-fold), with concordant effect directions.

#### 2.3.6 Bioinformatics analysis

Bioinformatics processing used Perseus (v1.15.7), Microsoft Excel, and R (v4.3.2). Differentially expressed proteins (DEPs) followed the predefined criteria (FDR < 0.05 and |log2 fold-change|≥0.263). Protein abundance matrices were clustered hierarchically, and functional annotation drew on UniProtKB/Swiss-Prot, KEGG, and Gene Ontology (GO).

GO/KEGG overrepresentation was tested by Fisher ‘ s exact test with FDR correction. GO terms were summarized by the three ontologies — biological process (BP), molecular function (MF), and cellular component (CC) —and enrichments were deemed significant at p < 0.05.

Protein -protein interaction networks were assembled with STRING (v4.3.0) and visualized in Cytoscape (v3.9. 1). Urinary creatinine levels were not measured in the current study.

### 2.4 Multiomics analysis

In this study, we performed a multiomics analysis to integrate metabolomic, proteomic, and transcriptomic data. The goal was to uncover correlations and associations between these datasets to identify key biomarkers for gastric cancer. Specifically, we used correlation analysis, principal component analysis (PCA), and other statistical methods to identify relationships between the different omic layers. For data integration, computational tools such as R and Python-based software packages were used. These methods allowed us to uncover enriched pathways and provide insights into the molecular mechanisms of gastric cancer. Additionally, pathway enrichment analysis was performed to identify key biological processes and signaling pathways relevant to gastric cancer progression.

## 3 Results

### 3.1 Subject characteristics and urine protein/metabolite detection

**Figure 1:**
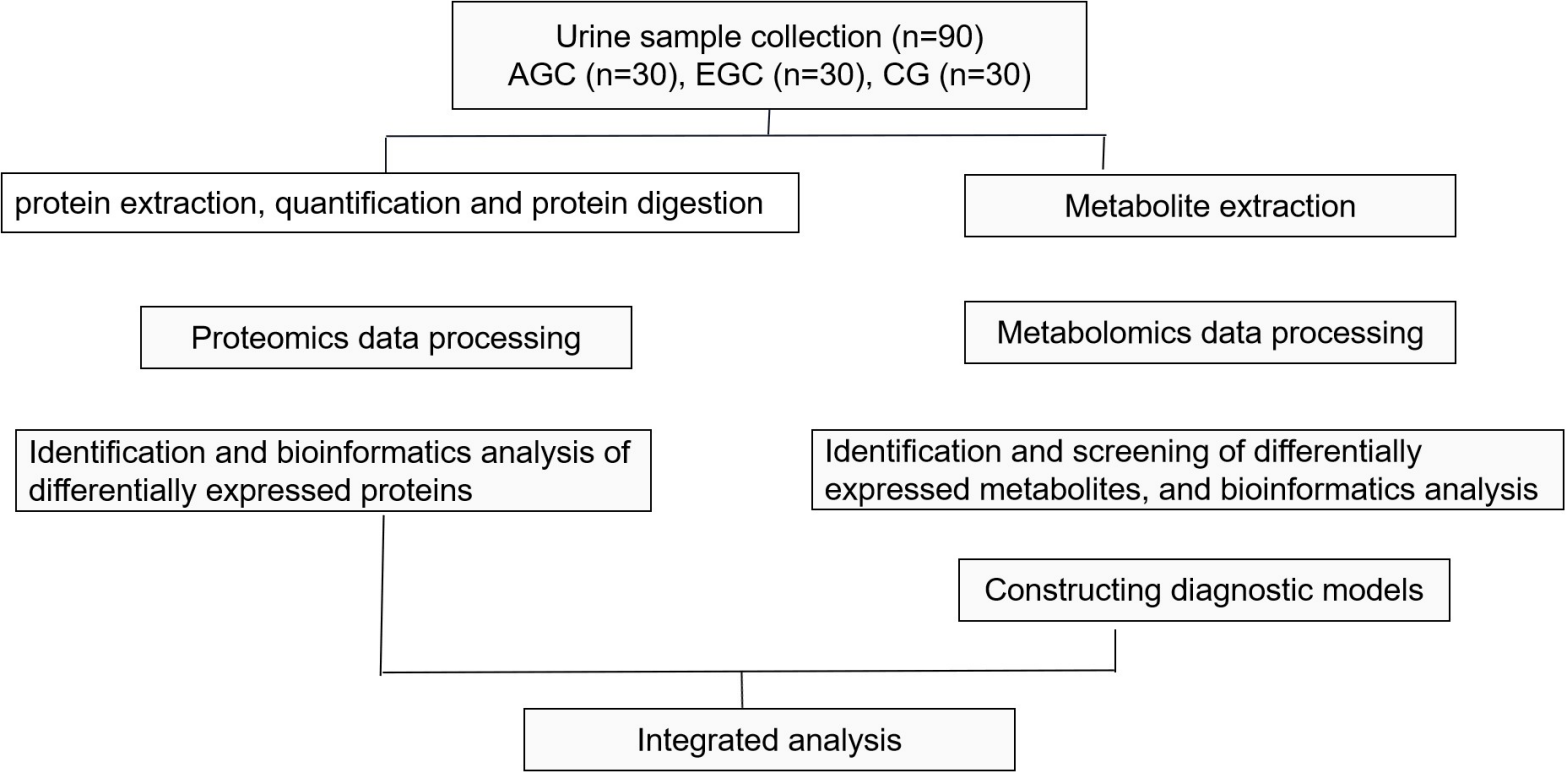
The schematic diagram of study design and work flow. GC, gastric cancer; EGC, early gastric cancer; CG, control group.

The study workflow is shown in **Figure 1**. Between September 2021 and May 2023, 90 participants were prospectively enrolled at two tertiary centers in China: Beijing Shijitan Hospital of Capital Medical University and Beijing Friendship Hospital. The participants included 30 with AGC, 30 with EGC, and 30 healthy controls (CG). For cancer cases, urine was collected before any therapy. Clinical and demographic features are listed in **Table 1**. Age and sex distributions did not differ significantly among groups, and the anatomical distribution of gastric lesions was similar between EGC and AGC (**Table 1**).

**Table 1:** Clinical sample summary of the participants included in this study.

Characteristics	AGC (n=30)	EGC (n=30)	CG (n=30)	*P*
Age (years)				0.196
Median (range)	64.5 (34-78)	65 (38-75)	63 (36-72)	
Sex				0.288
male	21	19	18	
female	9	11	12	
Location				0.360
fundus	6	7		
body	6	5		
antrum	18	18		

**Figure 2:**
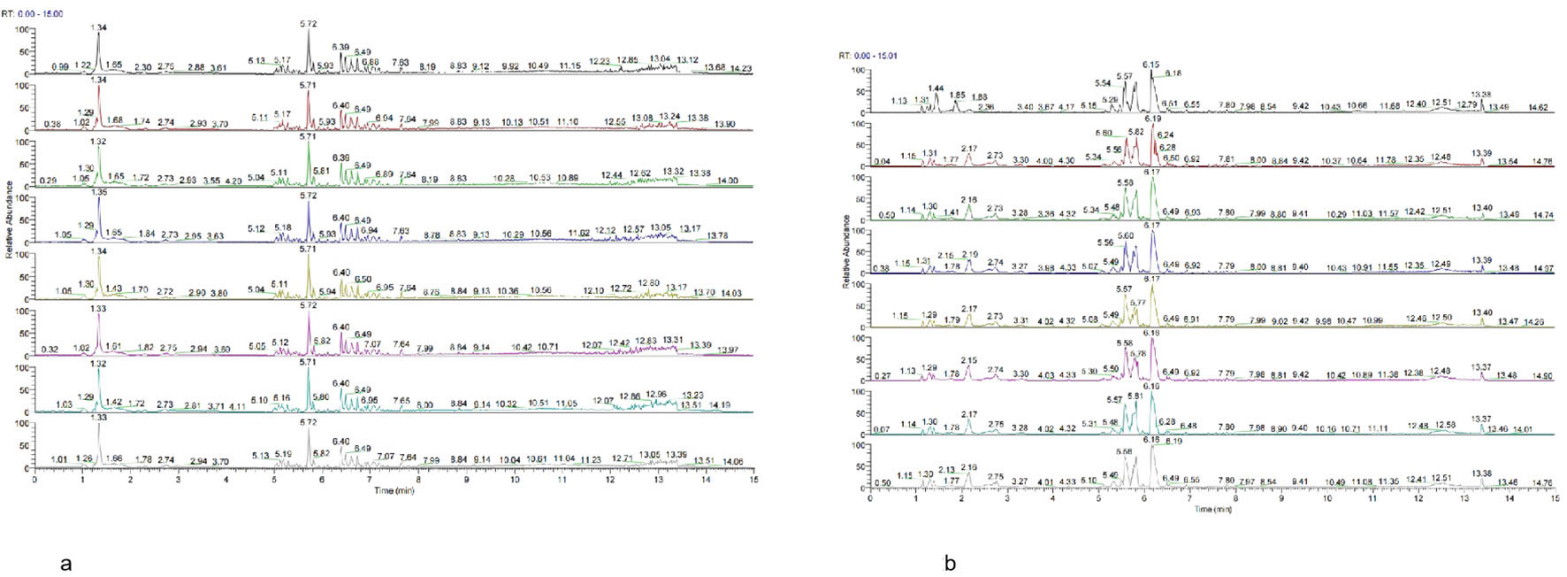
**(a)** Base peak chromatogram of QC samples in positive mode. **(b)** Base peak chromatogram of QC samples in negative mode.

**Figure 3:**
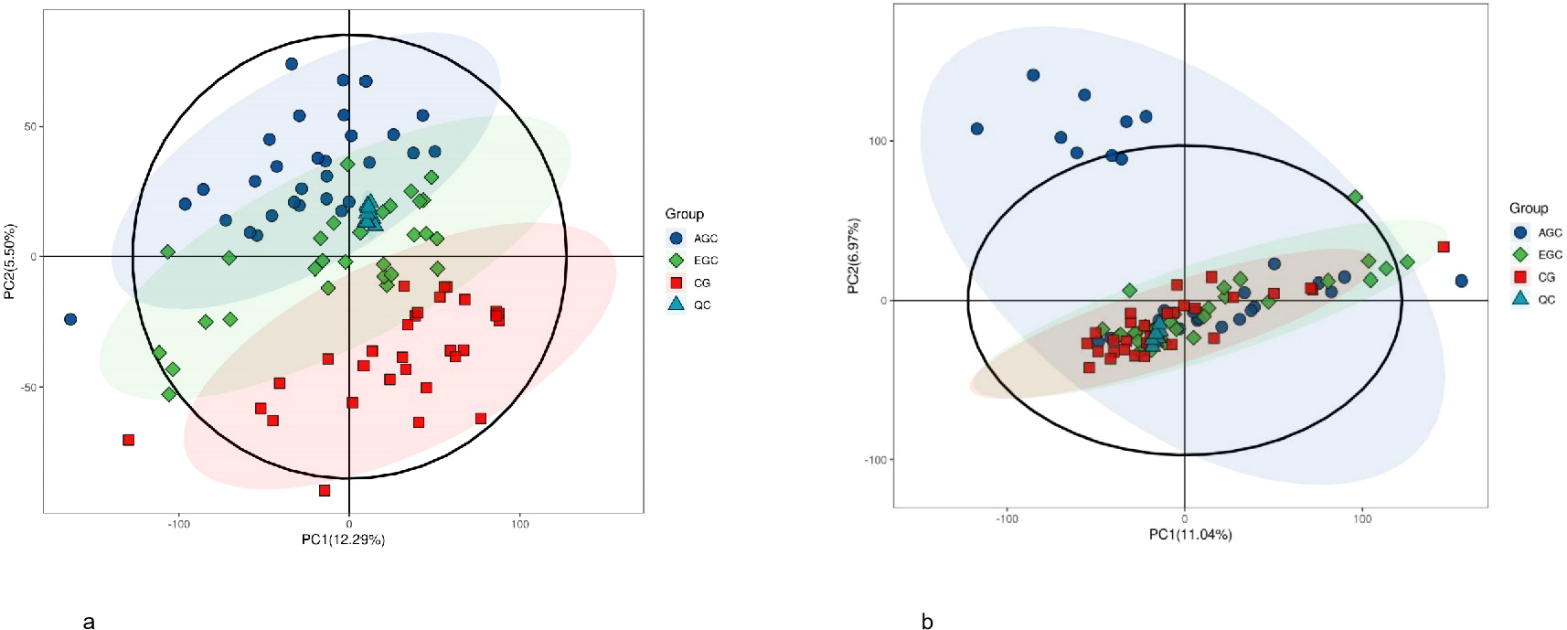
**(a)** Principal component analysis (PCA) of samples in positive mode. **(b)** Principal component analysis (PCA) of samples in positive mode.

To assess the stability of the experimental system, two strategies were employed in the metabolomics analysis: QC sample base peak comparison and principal component analysis (PCA) of the overall samples. The results showed consistent peak intensity and retention times across the QC samples, indicating minimal variation due to instrument errors and confirming reliable data quality (**Figure 2**). PCA analysis demonstrated that QC samples clustered tightly together, indicating good reproducibility of the experiment (**Figure 3**). Taken together, these results suggest that the analytical system in this study is stable, and the metabolic differences observed reflect the biological variations between the samples.

**Figure 4:**
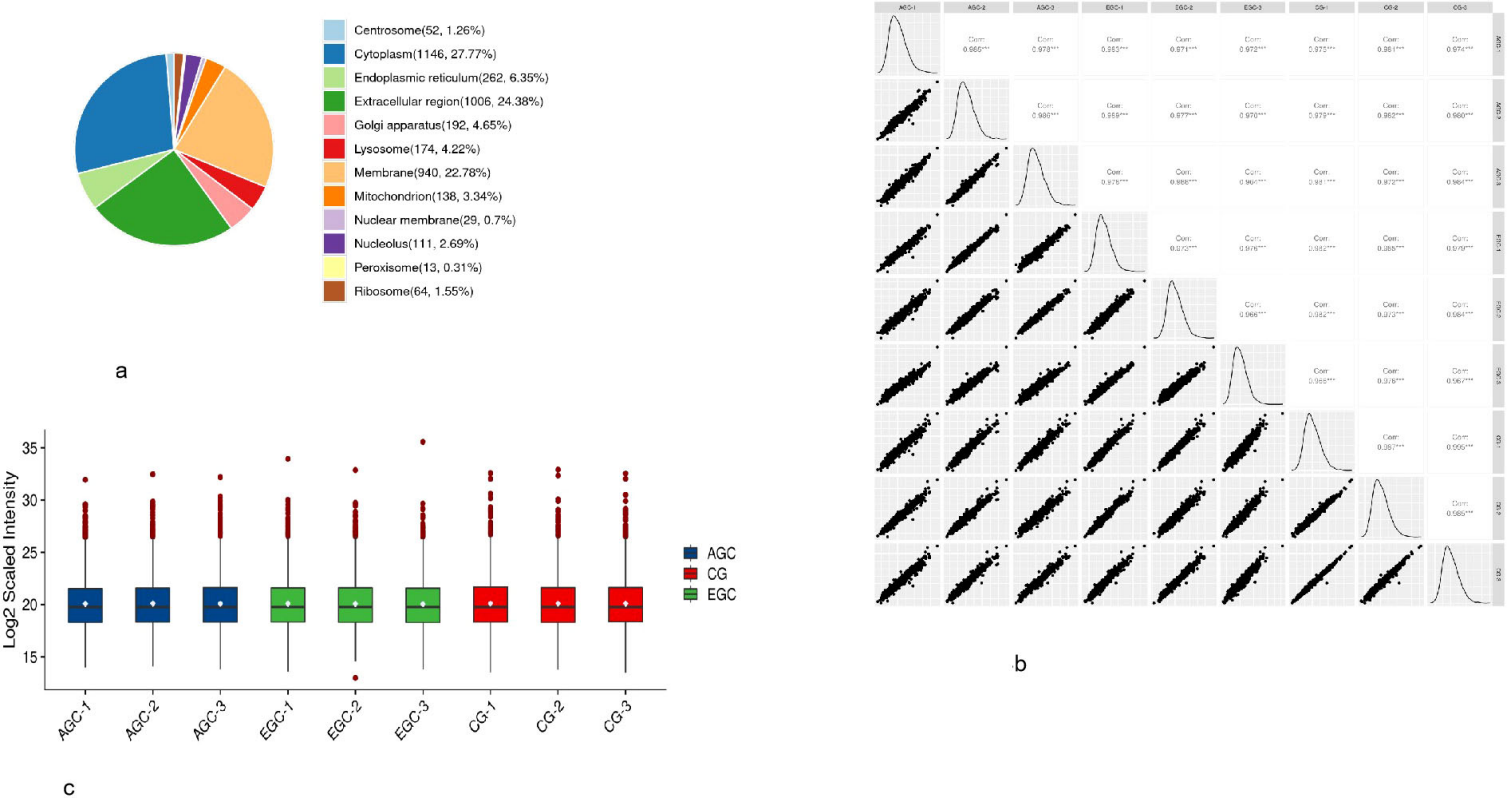
**(a)** Identification of protein cellular localization. **(b)** correlation heat map. **(c)** violin diagrams for all samples.

Across all samples, 2,506 proteins with unique peptide evidence were identified, of which 24.38% were located in the extracellular space (**Figure 4a**). LC - MS/MS quality-control runs showed excellent repeatability, with Spearman correlations of 0.97 - 0.99 (**Figure 4b**). Quantitative measurements across all urine samples were highly concordant, underscoring the robustness and stability of the urinary proteomics dataset (**Figure 4c**).

### 3.2 Urinary metabolite analysis

#### 3.2.1 Identification of differential metabolites between groups

In the metabolomics analysis, a total of 350 differential metabolites were identified in the comparison between advanced gastric cancer (AGC) and the control group (CG), while 285 differential metabolites were detected between early gastric cancer (EGC) and CG. Compared with the control group, 220 metabolites were downregulated and 130 were upregulated in the AGC group, whereas 201 metabolites were downregulated and 74 were upregulated in the EGC group. After excluding exogenous metabolites, 220 and 171 annotated differential metabolites from the AGC and EGC groups, respectively, were mapped to the Human Metabolome Database (HMDB) for Kyoto Encyclopedia of Genes and Genomes (KEGG) pathway enrichment and disease association analyses. These analyses were conducted to explore the potential biological functions and clinical relevance of the identified metabolic alterations in gastric cancer.

#### 3.2.2 HMDB classification of differential metabolites

**Figure 5:**
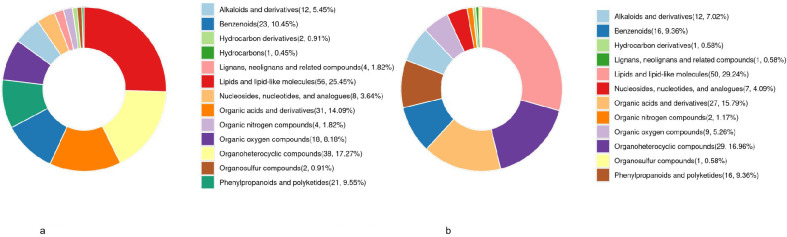
**(a)** Differential metabolite HMDB classification statistical analysis (AGC vs. CG). **(b)** Differential metabolite HMDB classification statistical analysis (EGC vs. CG).

Based on the Human Metabolome Database (HMDB) classification, significant compositional differences were observed between the AGC and EGC groups compared with the control group (CG). The altered metabolites were primarily distributed among lipids and lipid-like molecules, organic heterocyclic compounds, organic acids and their derivatives, and alkaloids and their derivatives (**Figure 5**). These results indicate that disruptions in lipid metabolism, amino acid turnover, and heterocyclic compound processing may play critical roles in the metabolic reprogramming associated with gastric cancer progression.

#### 3.2.3 KEGG pathway enrichment analysis

**Figure 6:**
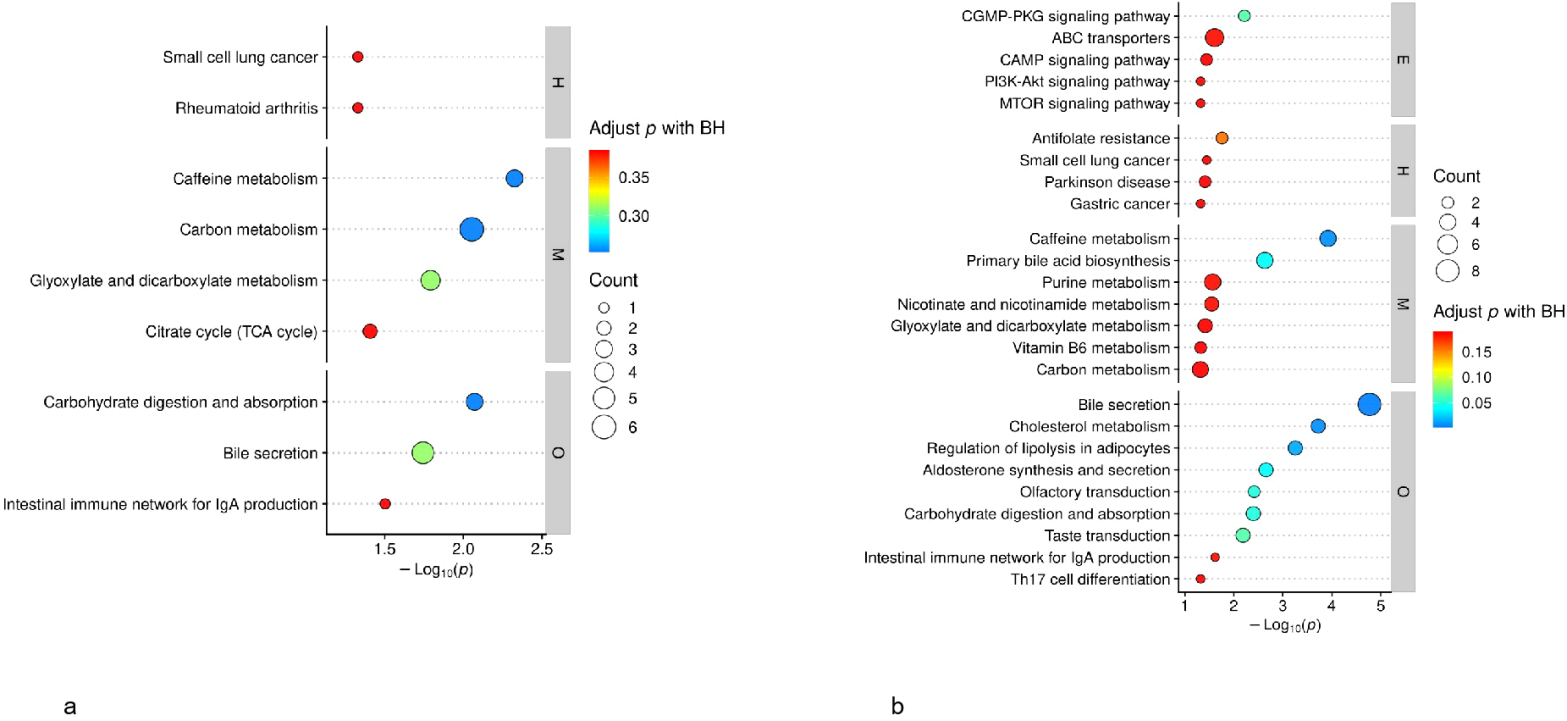
**(a)** Differential metabolite KEGG pathway analysis (AGC vs. CG). **(b)** Differential metabolite KEGG pathway analysis (EGC vs. CG).

Based on the KEGG pathway enrichment analysis, differential metabolites in advanced gastric cancer (AGC) compared with the control group (CG) were mainly involved in several key metabolic and immune pathways, including the tricarboxylic acid (TCA) cycle, glyoxylate and dicarboxylate metabolism, carbon metabolism, carbohydrate digestion and absorption, bile secretion, and the intestinal immune network for IgA production (**Figure 6a**).

In contrast, differential metabolites in early gastric cancer (EGC) compared with the control group (CG) were significantly enriched in both metabolic and signaling pathways, such as the PI3K-Akt signaling pathway, cAMP signaling pathway, cGMP-PKG signaling pathway, carbon metabolism, glyoxylate and dicarboxylate metabolism, purine metabolism, intestinal immune network for IgA production, carbohydrate digestion and absorption, lipid metabolism, cholesterol metabolism, and bile secretion (**Figure 6b**).

Collectively, these findings indicate that while both early and advanced stages of gastric cancer share core metabolic perturbations — particularly in carbon and amino acid metabolism — early-stage disease also exhibits activation of multiple signaling and immune pathways, suggesting a gradual metabolic -signaling transition during gastric tumor progression.

#### 3.2.4 Identification of key and core metabolites

To identify potential metabolite biomarkers for gastric cancer diagnosis, a weighted gene co-expression network analysis (WGCNA) was performed in addition to differential metabolite screening.

**Figure 7:**
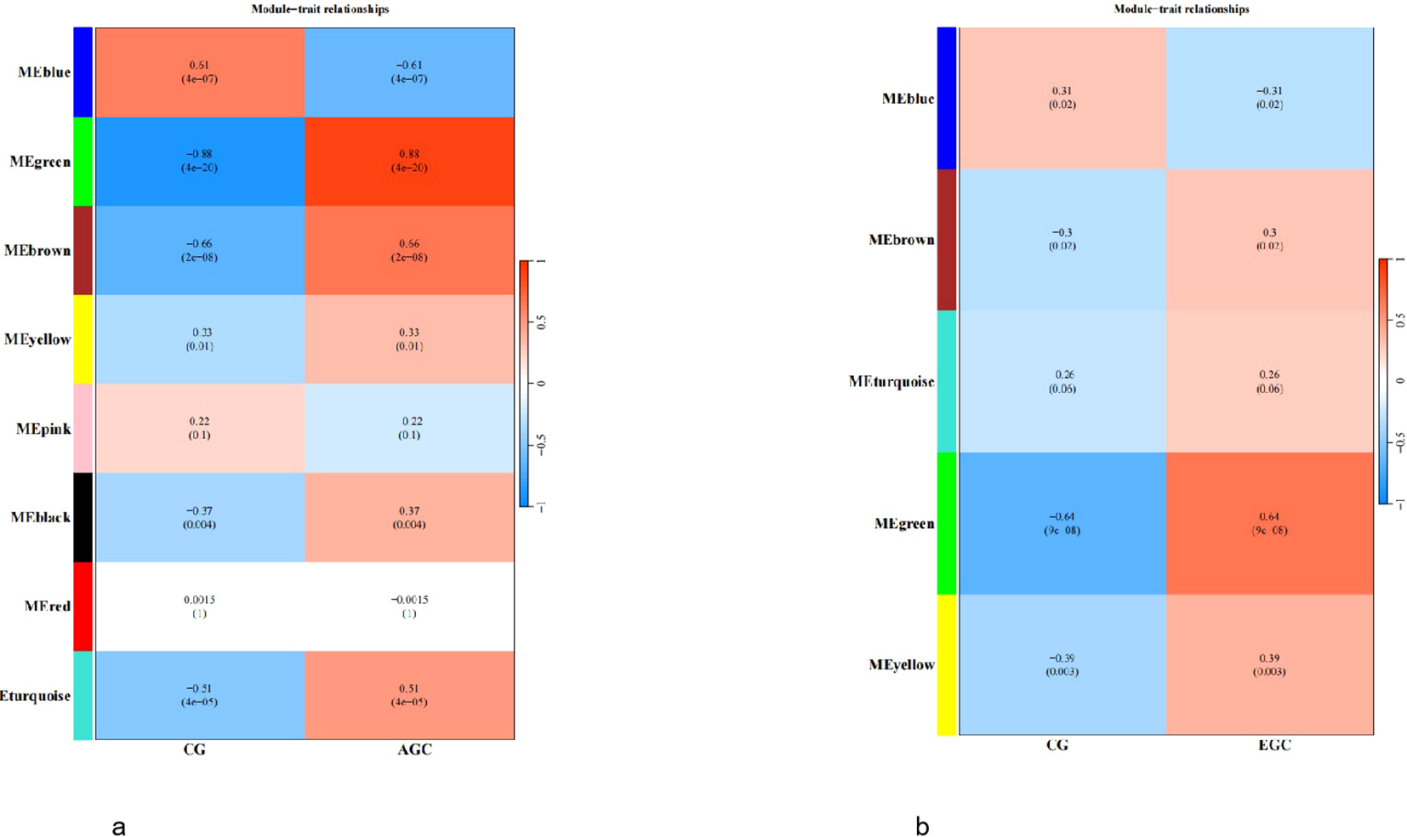
**(a)** WGCNA module heatmap (AGC vs. CG). Figure **(b)** WGCNA module heatmap (EGC vs. CG).

In the comparison between advanced gastric cancer (AGC) and the control group (CG), eight metabolite modules were identified. Correlation analysis between each module and clinical phenotypes revealed that the green, brown, and blue modules were highly associated with AGC (R = 0.88, 0.66, and 0.61; P = 4e-20, 2e-08, and 4e-07, respectively) (**Figure 7a**). In the comparison between early gastric cancer (EGC) and CG, five metabolite modules were identified, among which the green module showed a strong correlation with EGC (R = 0.64, P = 9e-08) (**Figure 7b**).

To determine key metabolites, the intersection of differential metabolites and module-specific metabolites from WGCNA was taken. This resulted in 57 key metabolites in AGC vs. CG and 16 key metabolites in EGC vs. CG.

**Figure 8:**
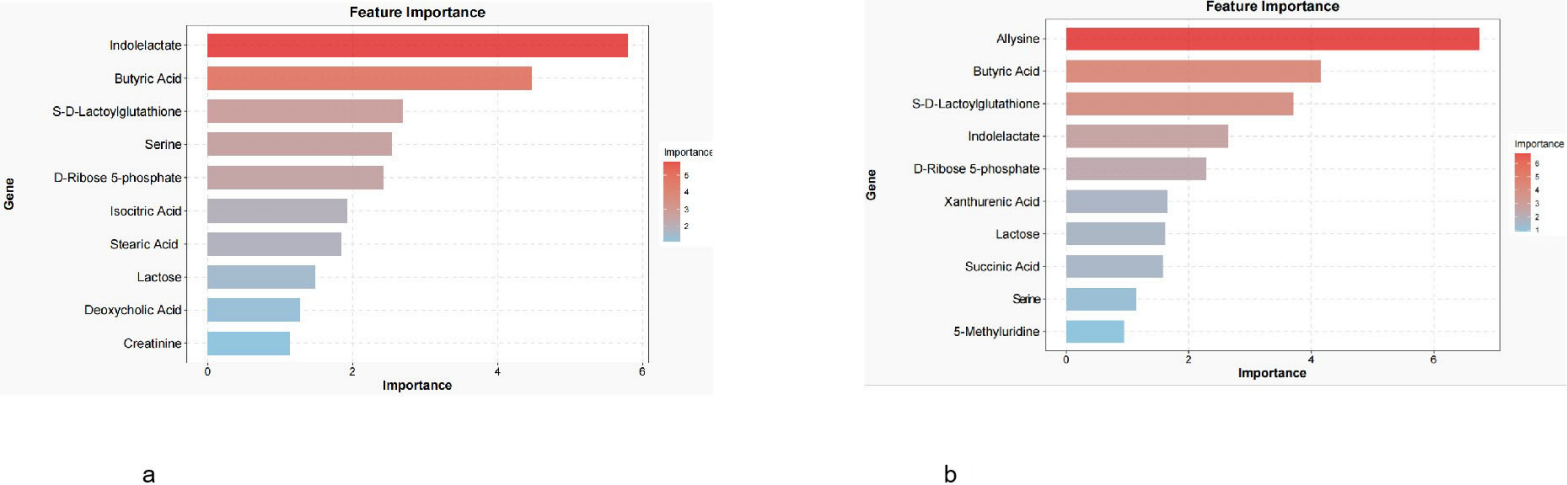
**(a)** Core differential metabolites (AGC vs. CG). **(b)** Core differential metabolites (EGC vs. CG).

For further refinement, the key metabolites were subjected to random forest and Boruta machine learning analyses to identify robust feature metabolites. The intersecting metabolites derived from both algorithms were defined as core metabolites for diagnostic modeling (**Figure 8**).

**Figure 9:**
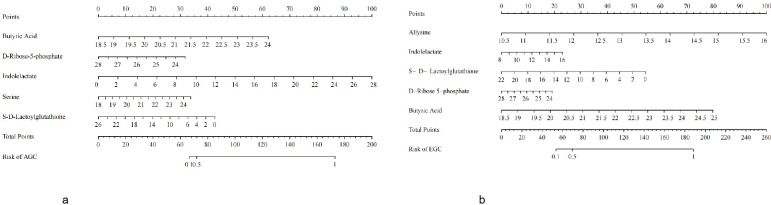
**(a)** Diagnostic model (AGC vs. CG). **(b)** Diagnostic model (AGC vs. CG).

Ultimately, the AGC diagnostic model included butyrate, D-ribose-5-phosphate, indolelactic acid, serine, and S-D-lactylglutathione (**Figure 9a**), while the EGC diagnostic model consisted of lysine, indolelactic acid, S-D-lactylglutathione, D-ribose-5-phosphate, and butyrate (**Figure 9b**). These metabolites demonstrated strong discriminatory power and potential utility as noninvasive biomarkers for early and advanced gastric cancer.

### 3.3 Urinary protein analysis

#### 3.3.1 Urine DEPs among three groups

**Figure 10:**
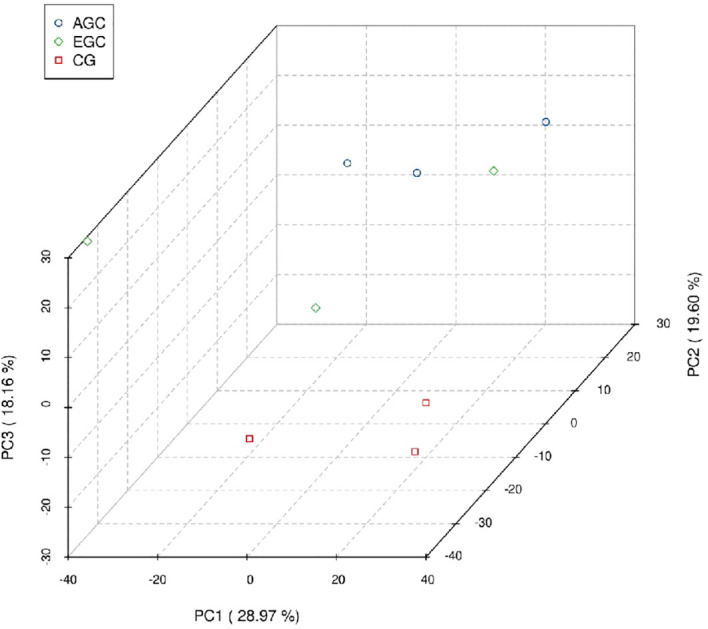
Principal component analysis (PCA).

Unsupervised principal component analysis (PCA) was used to examine global differences in urinary proteomes across groups. The score plot showed clear separation of CG from both cancer cohorts and a partial, yet evident, separation between EGC and AGC (**Figure 10**).

Relative to CG, the AGC cohort yielded 376 DEPs (100 upregulated, 276 downregulated), while the EGC cohort had 191 DEPs (35 upregulated, 156 downregulated). Direct AGC -EGC comparison identified 92 DEPs (53 upregulated, 39 downregulated).

**Figure 11:**
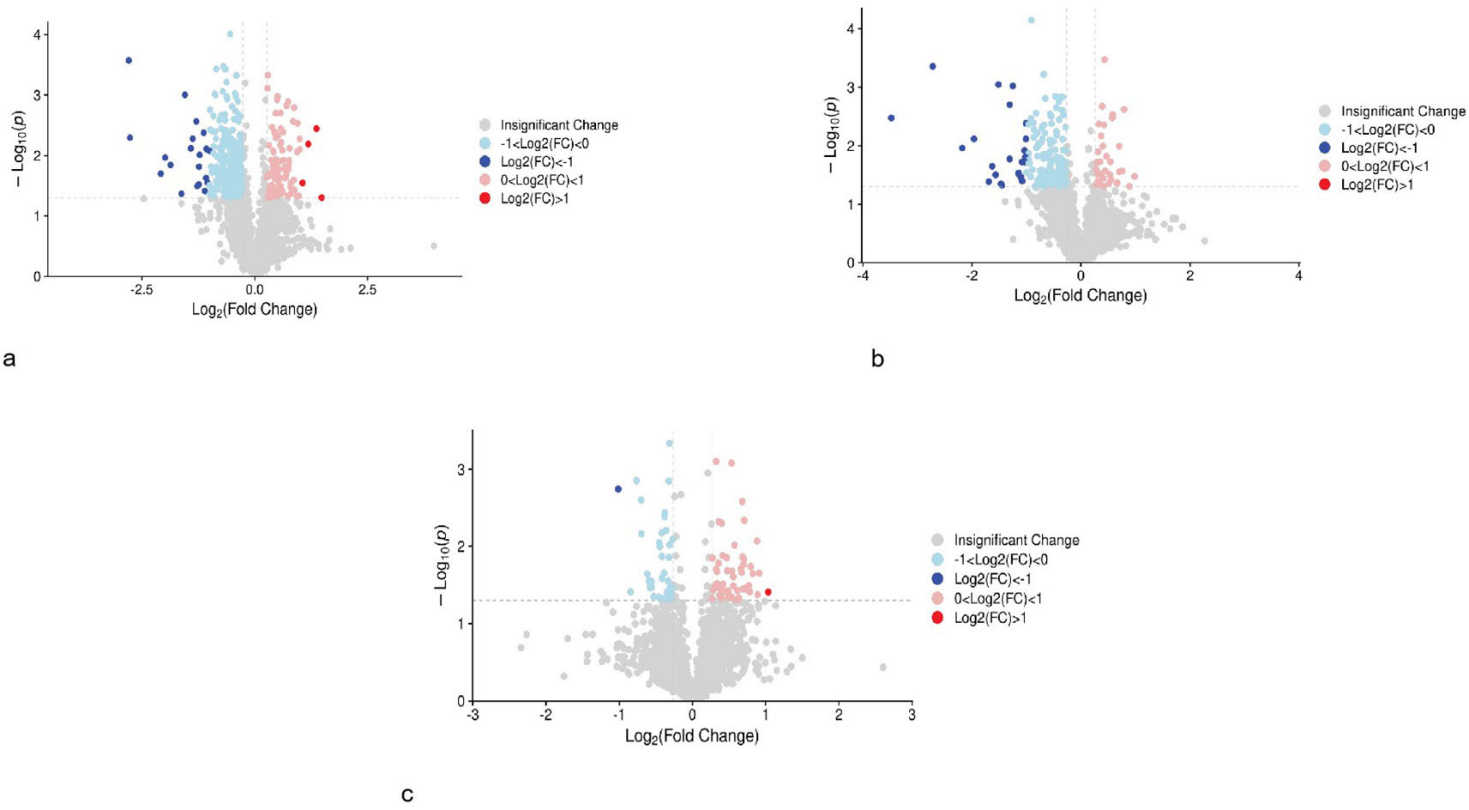
**(a)** Quantitative volcano plot of DEP in AGC/CG group. **(b)** Quantitative volcano plot of DEP in EGC/CG group. **(c)** Quantitative volcano plot of DEP in AGC/EGC group.

Volcano plots highlight proteins with fold change >1.2 and p < 0.05 in red (significant upregulation) and those with fold change <0.83 and p < 0.05 in blue (significant downregulation); nonsignificant proteins appear in grey (**Figure 11**). The top 10 most increased and decreased proteins are listed in **Table 2**.

**Table 2:** List of differentially expressed proteins.

Protein accessions	Protein descriptions	Gene name	FDR	FC	p Value
Up-regulated					
E9PK54	Heat shock cognate 71 kDa protein	HSPA8	0.335	1.341	0.0044
C0JYZ2	Titin	TTN	0.539	1.327	0.036
J3QT83	Collagen alpha-1(XIV) chain (Fragment)	COL14A1	0.532	1.445	0.031
B4E324	cDNA FLJ60397, highly similar to Lysosomal protective protein	CTSA	0.335	1.350	0.00034
A5PLM9	Cathepsin L1	CTSL	0.335	1.311	0.0021
A0A024R7F4	Deoxyribonuclease II	DNASE2	0.439	1.625	0.010
P54886	Delta-1-pyrroline-5-carboxylate synthase	ALDH18A1	0.586	1.853	0.049
Q6S4P3	Ferritin	FTL	0.335	1.488	0.0033
P21399	Cytoplasmic aconitate hydratase	ACO1	0.583	1.274	0.046
P11216	Glycogen phosphorylase, brain form	PYGB	0.140	1.225	0.00047
Q86Y22	Collagen alpha-1(XXIII) chain	COL23A1	0.140	1.671	0.0013
Q9NP84	Tumor necrosis factor receptor superfamily member 12A	TNFRSF12A	0.164	1.806	0.0027
A0A140VK46	Proteasome subunit beta	PSMB4	0.171	1.302	0.0034
F2X0U9	Truncated CD61 (Fragment)	ITGB3	0.172	2.280	0.0065
A0A0A0MT01	Gelsolin	GSN	0.188	1.700	0.012
P02452	Collagen alpha-1(I) chain	COL1A1	0.190	1.493	0.012
P13671	Complement component C6	C6	0.228	1.364	0.024
B1AKG0	Complement factor H-related protein 1	CFHR1	0.266	1.384	0.037
C9J9S3	Serine/threonine-protein phosphatase (Fragment)	PPP1CB	0.312	1.241	0.038
Down-regulated					
Q09666	Neuroblast differentiation-associated protein AHNAK	AHNAK	0.359	0.549	0.0053
A1A4E9	Keratin 13	KRT13	0.584	0.367	0.048
B7ZMD7	Alpha-amylase	AMY1A	0.426	0.601	0.0088
P29508	Serpin B3	SERPINB3	0.335	0.636	0.0016
P12109	Collagen alpha-1(VI) chain	COL6A1	0.461	0.679	0.013
V9HWN7	Fructose-bisphosphate aldolase	ALDOA	0.532	0.770	0.024
A0A5F9ZHM4	L-lactate dehydrogenase	LDHB	0.572	0.719	0.045
P06733	Alpha-enolase	ENO1	0.335	0.685	0.0031
P48594	Serpin B4	SERPINB4	0.488	0.736	0.015
P04792	Heat shock protein beta-1	HSPB1	0.509	0.513	0.020
P15924	Desmoplakin	DSP	0.140	0.686	0.000098
P07355	Annexin A2	ANXA2	0.140	0.619	0.00033
Q92859	Neogenin	NEO1	0.140	0.650	0.00061
Q6P2Q9	Pre-mRNA-processing-splicing factor 8	PRPF8	0.140	0.747	0.0010
Q02388	Collagen alpha-1(VII) chain	COL7A1	0.140	0.656	0.0012
P53990	IST1 homolog	IST1	0.155	0.707	0.0016
Q9NYB9	Abl interactor 2	ABI2	0.158	0.632	0.0021
O75144	ICOS ligand	ICOSLG	0.158	0.598	0.0022
Q5JXB2	Putative ubiquitin-conjugating enzyme E2 N-like	UBE2NL	0.158	0.530	0.0022
A0A024R326	60S ribosomal protein L29	RPL29	0.164	0.728	0.00257

#### 3.3.2 GO and KEGG annotation analyses of the differentially expressed proteins

To contextualize the DEPs, we performed GO and KEGG overrepresentation analyses, summarizing GO terms across biological process (BP), cellular component (CC), and molecular function (MF).

Versus CG, the AGC cohort was enriched for BP terms related to cell/biological adhesion and negative regulation of biological processes; CC terms mapped mainly to extracellular membrane–bounded organelles and exosomes; MF terms clustered in cell-adhesion molecule binding, structural molecule activity, and cadherin binding (**Supplementary Figure 1a**). KEGG analysis (p < 0.05) highlighted 11 pathways, notably cell adhesion molecules (CAMs), ECM–receptor interaction, platelet activation, complement/coagulation cascades, and 2-oxocarboxylic acid metabolism (**Supplementary Figure 2a**).

Relative to CG, the EGC cohort showed BP enrichment for negative regulation of biological processes and immune response; CC terms again centered on extracellular membrane–bounded organelles and exosomes; MF terms overlapped with cell-adhesion molecule binding, structural molecule activity, and cadherin binding (**Supplementary Figure 1b**). KEGG enrichment (p < 0.05) identified 11 pathways, including HIF-1 signaling, glycolysis/gluconeogenesis, amino-acid biosynthesis, antigen processing/presentation, and dicarboxylate metabolism (**Supplementary Figure 2b**).

Together, these annotations implicate adhesion, extracellular vesicle biology, and metabolic/signaling programs as central features of GC pathobiology, providing a framework for future mechanistic studies.

### 3.4 Combined protein and metabolite analysis

An integrated multi-omics analysis combining proteomics and metabolomics was performed to uncover convergent molecular pathways underlying gastric cancer progression.

In the AGC vs. CG comparison, KEGG enrichment results from differential proteins and metabolites were intersected using a Venn analysis, revealing 43 overlapping pathways shared between the two omics datasets (**Supplementary Figure 3a**). Visualization using a joint bubble plot highlighted several key pathways (**Supplementary Figure 3b**), including amino acid metabolism, carbon metabolism, and the tricarboxylic acid (TCA) cycle, which are central to cancer cell energy homeostasis and biosynthetic regulation.

Similarly, in the EGC vs. CG comparison, Venn analysis identified 30 shared KEGG pathways enriched in both proteomic and metabolomic datasets (**Supplementary Figure 3c**). Notably, these overlapped pathways were predominantly involved in amino acid metabolism, carbon metabolism, PI3K -Akt signaling, and cellular immune responses (**Supplementary Figure 3d**), suggesting coordinated dysregulation of metabolic and signaling networks during the early stages of gastric tumorigenesis.

**Figure 12:**
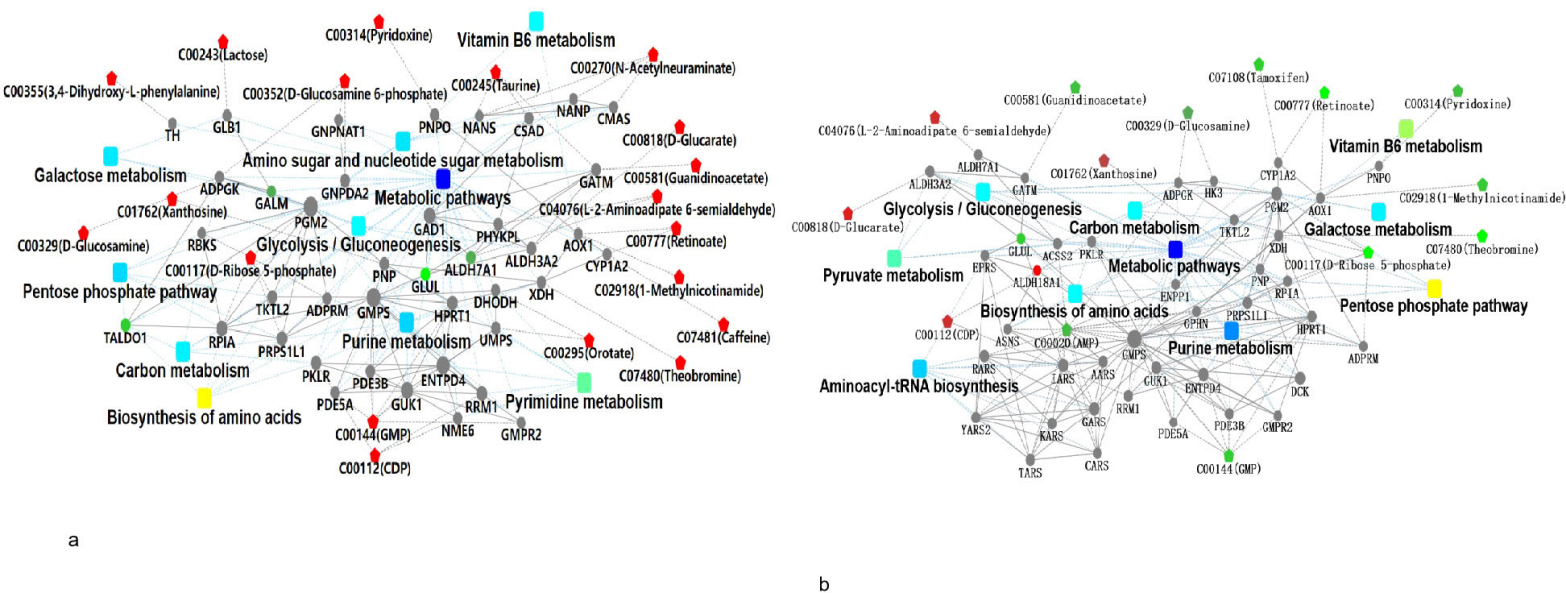
**(a)** Metabolomics and proteomics integrated analysis network diagram (AGC vs. CG). **(b)** Metabolomics and proteomics integrated analysis network diagram (EGC vs. CG).

To further elucidate molecular interactions, a protein - metabolite - pathway network was constructed (**Figure 12**). This network analysis revealed several proteins and metabolites occupying hub positions that bridged multiple pathways, implying their central regulatory roles in metabolic reprogramming. Core metabolites and their corresponding proteins were consistently identified across the key pathways, providing mechanistic insights into the metabolic - proteomic crosstalk driving gastric cancer development.

## 4 Discussion

This study integrates urinary metabolomics and proteomics to characterize molecular alterations across early (EGC) and advanced gastric cancer (AGC) and to develop non-invasive candidate biomarker panels. In line with the increasing recognition of urine as an accessible biofluid for cancer biomarker discovery, our findings further support the feasibility of urine-based multi-omics profiling for GC detection and biological interpretation. Previous studies have identified urine proteomic signatures associated with gastric lesion progression (12, 14), as well as metabolomics-based predictive models for GC detection (13, 16), providing a contextual framework for our integrative approach.

### 4.1 Principal findings and interpretation

We observed robust separation among controls, EGC, and AGC at both the metabolite and protein levels under stringent quality control, identifying 285 differential metabolites and 191 differential proteins in EGC versus controls, and 350 metabolites and 376 proteins in AGC versus controls. These stage-stratified molecular changes indicate that gastric tumorigenesis is accompanied by progressive systemic metabolic and host-response remodeling detectable in urine (16, 17).

A key finding is the stage-dependent shift in pathway patterns. In AGC, enriched pathways were dominated by core metabolism (e.g., TCA cycle, carbon metabolism, bile secretion), consistent with extensive metabolic reprogramming in advanced disease. In contrast, EGC showed additional enrichment of signaling and immune-related pathways (e.g., PI3K–Akt, cAMP/cGMP–PKG signaling, intestinal IgA network), suggesting that early disease may be characterized by dysregulated host–tumor interaction and signaling activation before overt late-stage metabolic collapse (13, 18).

Machine-learning refinement of WGCNA-derived metabolites yielded compact panels for AGC and EGC diagnosis (shared markers such as butyrate, D-ribose-5-phosphate, indolelactic acid, and S-D-lactylglutathione, with stage-specific differences). These results support the concept that a small metabolite signature can capture clinically relevant GC-associated metabolic states and may be deployable for non-invasive screening after targeted validation (19).

Importantly, multi-omics intersection analysis identified overlapping pathways between proteomics and metabolomics (43 shared pathways in AGC; 30 in EGC), highlighting coordinated dysregulation rather than isolated single-omic changes. The hub protein–metabolite–pathway topology further suggests that a subset of molecules may act as cross-pathway connectors, providing a mechanistic rationale for selecting a small number of candidate hub proteins for downstream validation (20, 21).

### 4.2 Biological implications

Beyond pathway-level summaries, our data imply biologically meaningful differences between early and advanced disease. The relative prominence of signaling/immune pathways in EGC may reflect early remodeling of the tumor microenvironment and mucosal immunity, whereas the strong metabolic signatures in AGC are consistent with increased energetic and biosynthetic demand, systemic inflammation, and broader tissue disruption as invasion progresses. This interpretation aligns with the view that multi-omics integration is valuable for capturing both tumor-intrinsic metabolic rewiring and host-response programs across stages (22, 23).

From the proteomic side, several candidate hub proteins identified in our networks are biologically plausible in the context of the enriched KEGG categories observed here (e.g., CAMs/ECM interaction, platelet activation, antigen processing, HIF-1/glycolysis programs). For example, TNFRSF12A (Fn14) is a TNF receptor family member implicated in pro-inflammatory signaling and has been linked to invasive phenotypes in several cancers (24); ITGB3 participates in cell–ECM adhesion and can connect extracellular matrix remodeling with platelet-related and metastatic processes (25); HSPA8 is a central chaperone involved in proteostasis and stress adaptation, and has been shown to promote gastric cancer progression by activating the canonical Wnt signaling pathway and glycolysis (26); CTSL is a lysosomal protease with potential roles in extracellular matrix degradation and invasion (27); FTL is involved in iron storage and may interface with oxidative stress and iron-dependent cell-death susceptibility, and its expression has been associated with tumor progression and poor prognosis in gastric cancer (28). Collectively, these proteins provide a coherent biological narrative consistent with our enrichment patterns and support prioritizing them for targeted assays and mechanistic follow-up.

### 4.3 Clinical implications

Urine-based multi-omics offers a practical route toward non-invasive GC detection, with advantages in accessibility, patient acceptability, and suitability for longitudinal sampling. The metabolite panels identified here may support early detection and risk stratification, potentially complementing endoscopy in pre-screening or surveillance settings. In parallel, the candidate hub proteins (TNFRSF12A, ITGB3, HSPA8, CTSL, FTL) may serve as a focused set for targeted verification, especially given that several appear extracellular/vesicle-associated, which is compatible with urinary detectability. Crucially, early- versus late-stage markers are not interchangeable. Molecules associated with EGC are more likely to reflect early host–tumor signaling and immune perturbations, whereas AGC-associated signals may reflect extensive metabolic derangement and invasion-related remodeling. Therefore, for clinical translation, stage-aware modeling (e.g., separate EGC-focused screening panels and AGC-focused burden/progression markers) should be considered, particularly if the intended application is early diagnosis rather than general case–control separation.

Our findings are broadly consistent with prior integrative multi-omics studies highlighting coordinated metabolic and signaling alterations in gastric cancer (29, 30). In addition, Shen et al. provided a comprehensive review of the emerging role of multi-omics strategies in precision diagnosis and treatment of GC (31).

### 4.4 Strengths and limitations

This study has several strengths. First, the dual-center design reduces center-specific bias and improves the generalizability of the observed urine multi-omics signatures. Second, paired metabolomics and proteomics were generated from the same biospecimens under rigorous quality control, enabling pathway-level cross-validation across omic layers. Third, the combination of WGCNA and machine-learning feature selection provides a transparent strategy to reduce high-dimensional signals into compact candidate panels suitable for downstream targeted assays. Several limitations should also be acknowledged. The sample size (n=90) is moderate and requires confirmation in larger, demographically diverse cohorts, ideally including external prospective validation. In addition, urinary signals can be influenced by renal function, hydration status, and systemic comorbidities; although total protein normalization was applied, the absence of creatinine-based normalization may introduce inter-individual variability and should be addressed in future studies. Finally, targeted validation (e.g., PRM/MRM for proteins and targeted LC–MS for metabolites) and functional experiments were not performed here; thus, the proposed hub molecules should be interpreted as high-priority candidates rather than definitive mechanistic drivers.

### 4.5 Future directions

Future work should prioritize independent validation in external cohorts, longitudinal sampling to evaluate biomarker dynamics over time, and functional studies to clarify how key metabolites and proteins contribute to GC biology. Integrating additional data types (e.g., imaging, transcriptomics, microbiome profiles) may further improve stage-aware predictive models and enhance clinical interpretability. Translational efforts focused on developing readily deployable urine-based assays (e.g., targeted MS panels or immunoassays) will be crucial for clinical application (32).

## 5 Conclusion

This study demonstrates the potential of urine-based multi-omics biomarkers for the diagnosis of gastric cancer, providing valuable insights into the metabolic and proteomic alterations driving cancer progression. The identified core metabolites and proteins could serve as promising candidates for non-invasive diagnostic tools, with the potential for clinical application in early-stage screening and risk stratification. Future studies should validate these findings and explore their clinical utility in larger, more diverse cohorts.

## Data Availability

The data presented in the study are deposited in Dryad, https://doi.org/10.5061/dryad.zkh1893rf.
