# Supplementary material for: Urinary metabolomics and proteomics for early detection of gastric cancer: insights from a two-center multicenter study
Source: Front Oncol. 2026 Apr 1;16:1733804. doi: 10.3389/fonc.2026.1733804 (PMC13081149; doi:10.3389/fonc.2026.1733804)
Supplement: Supplementary file 1 [file Presentation1.pptx]

## Slide 1
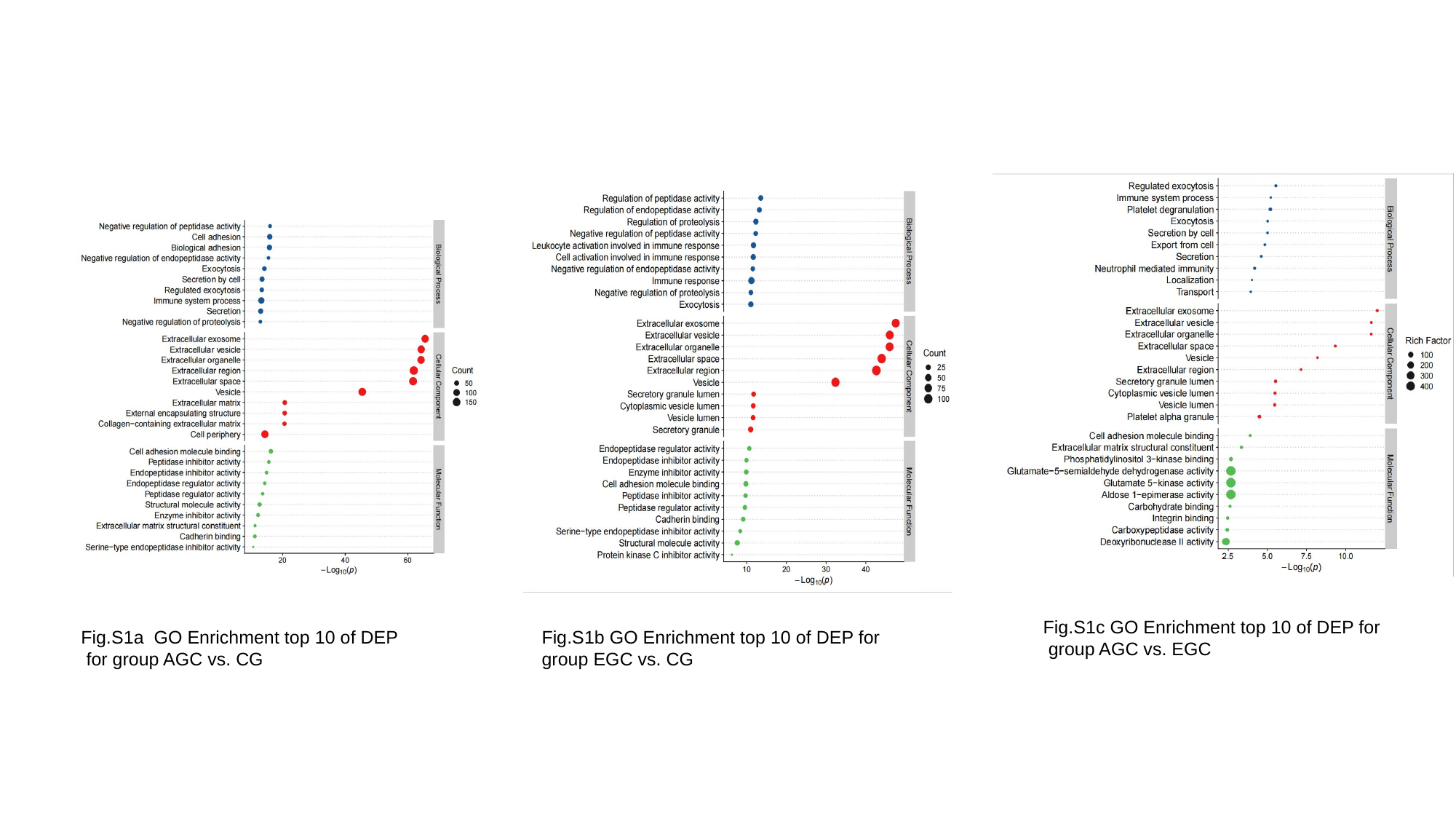

Fig.S1c GO Enrichment top 10 of DEP for
 group AGC vs. EGC
Fig.S1a GO Enrichment top 10 of DEP
 for group AGC vs. CG
Fig.S1b GO Enrichment top 10 of DEP for
group EGC vs. CG

## Slide 2
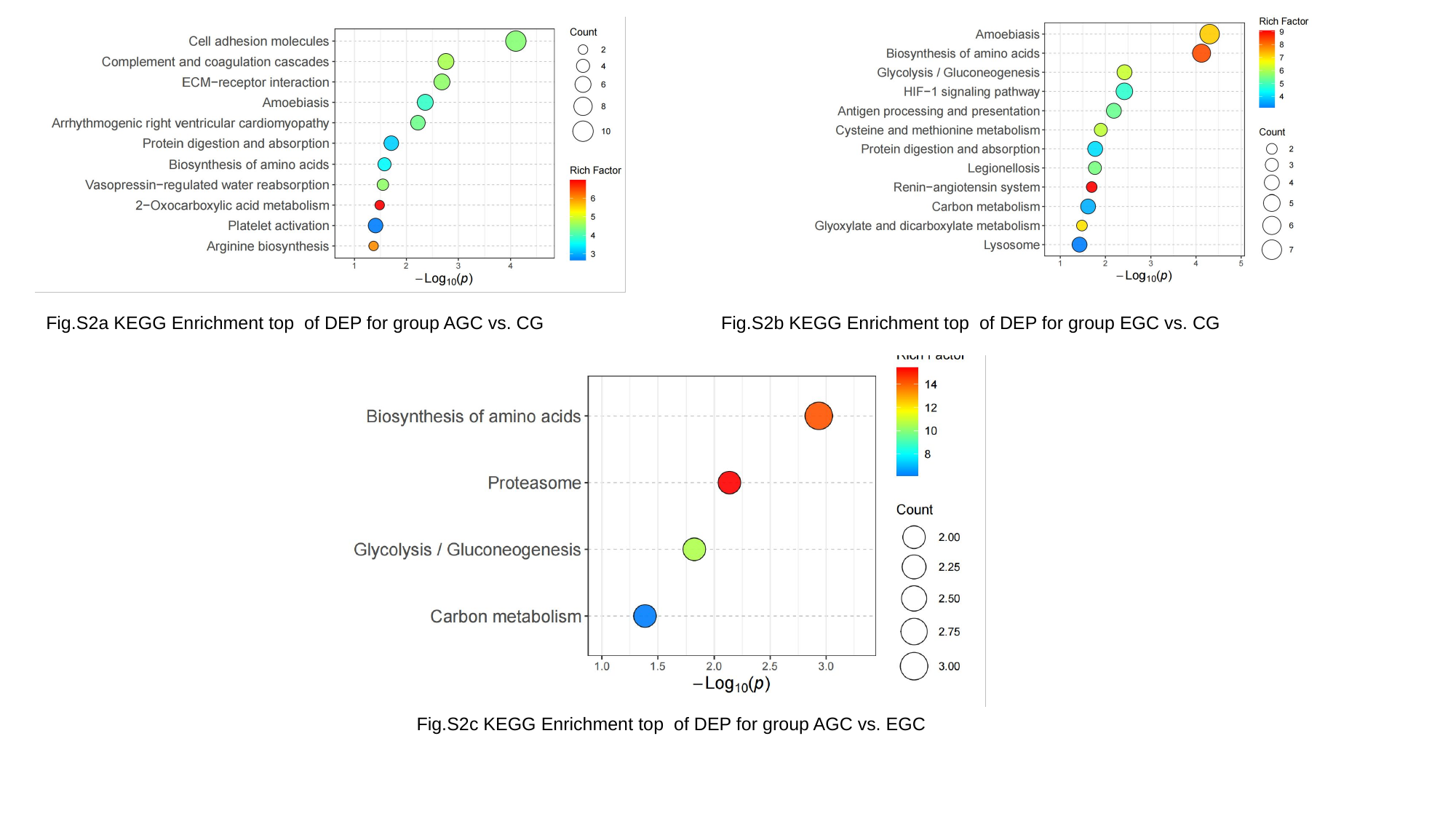

Fig.S2a KEGG Enrichment top of DEP for group AGC vs. CG
Fig.S2b KEGG Enrichment top of DEP for group EGC vs. CG
Fig.S2c KEGG Enrichment top of DEP for group AGC vs. EGC

## Slide 3
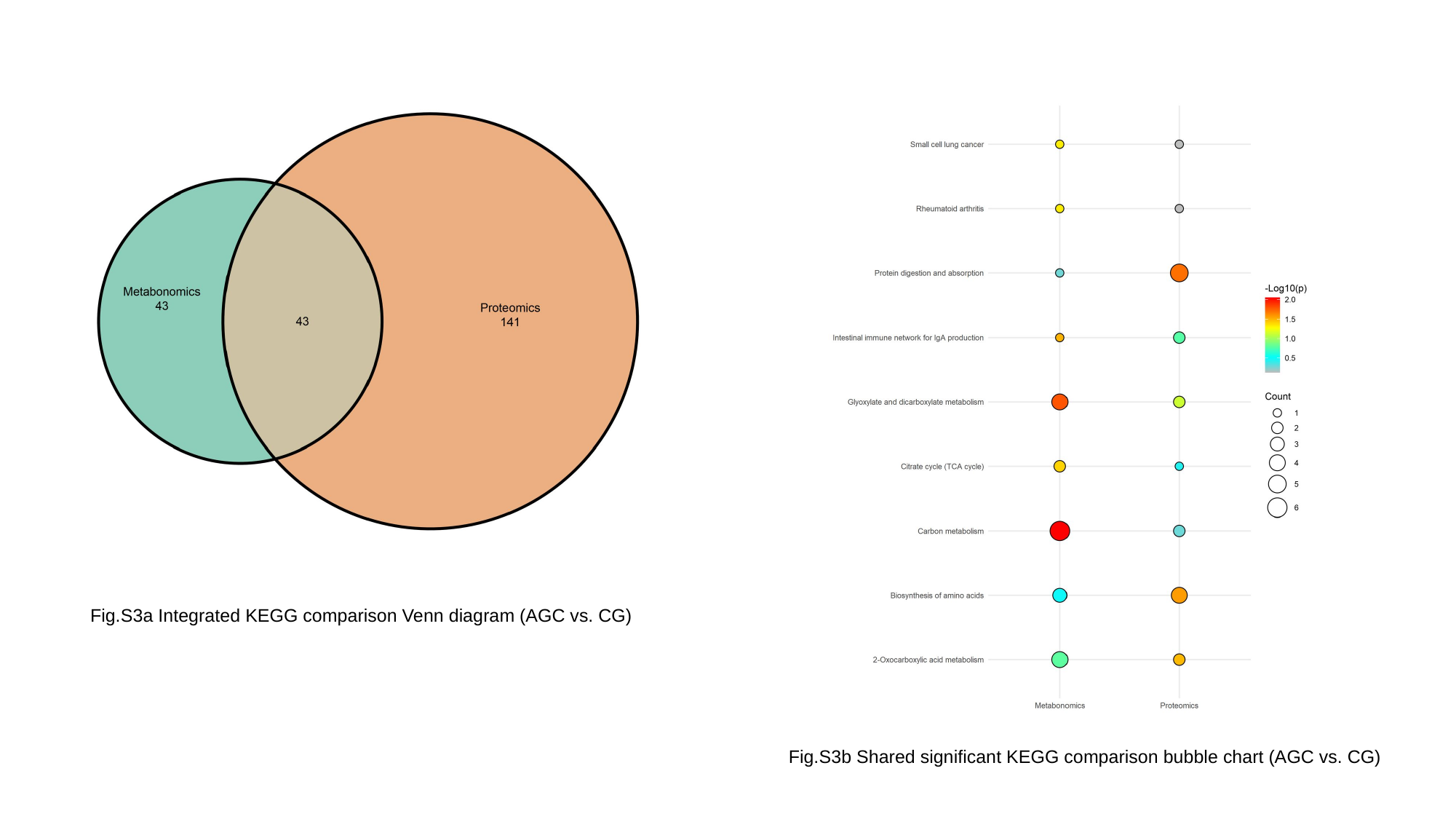

Fig.S3a Integrated KEGG comparison Venn diagram (AGC vs. CG)
Fig.S3b Shared significant KEGG comparison bubble chart (AGC vs. CG)

## Slide 4
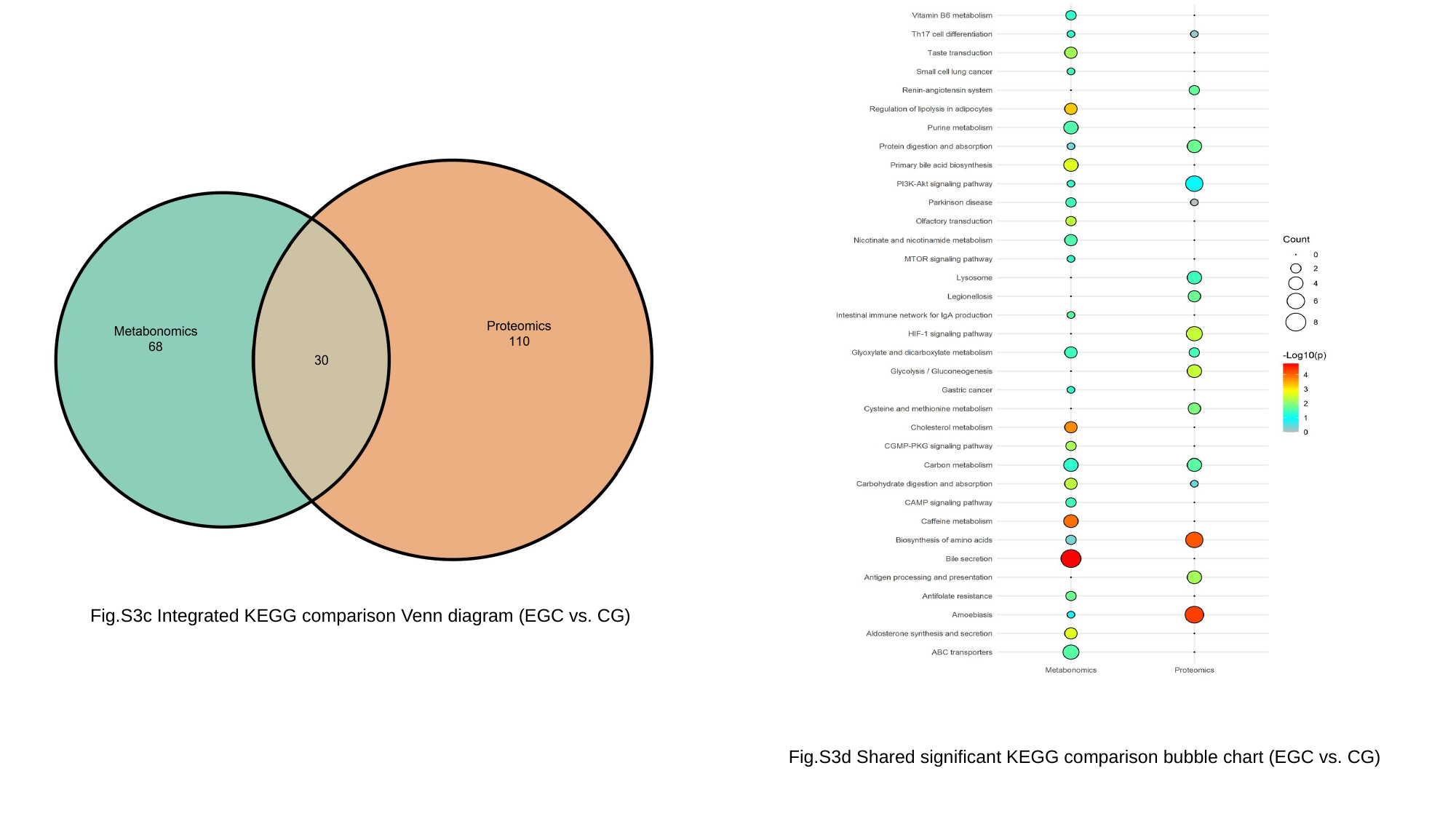

Fig.S3c Integrated KEGG comparison Venn diagram (EGC vs. CG)
Fig.S3d Shared significant KEGG comparison bubble chart (EGC vs. CG)
